# The Challenge of Stability in High-Throughput Gene Expression Analysis: Comprehensive Selection and Evaluation of Reference Genes for BALB/c Mice Spleen Samples in the *Leishmania infantum* Infection Model

**DOI:** 10.1371/journal.pone.0163219

**Published:** 2016-09-26

**Authors:** Yasmina E. Hernandez-Santana, Eduardo Ontoria, Ana C. Gonzalez-García, M. Antonieta Quispe-Ricalde, Vicente Larraga, Basilio Valladares, Emma Carmelo

**Affiliations:** 1 Instituto Universitario de Enfermedades Tropicales y Salud Pública de Canarias, Universidad de la Laguna, La Laguna, Islas Canarias, Spain; 2 Departamento de Biología, Facultad de Ciencias, Universidad Nacional de San Antonio Abad del Cusco, Cusco, Peru; 3 Laboratorio de Parasitología Molecular, Departamento de Microbiología Molecular y Biología de las Infecciones, Centro de Investigaciones Biológicas, Consejo Superior de Investigaciones Científicas, Madrid, Spain; Northwestern University, UNITED STATES

## Abstract

The interaction of *Leishmania* with BALB/c mice induces dramatic changes in transcriptome patterns in the parasite, but also in the target organs (spleen, liver…) due to its response against infection. Real-time quantitative PCR (qPCR) is an interesting approach to analyze these changes and understand the immunological pathways that lead to protection or progression of disease. However, qPCR results need to be normalized against one or more reference genes (RG) to correct for non-specific experimental variation. The development of technical platforms for high-throughput qPCR analysis, and powerful software for analysis of qPCR data, have acknowledged the problem that some reference genes widely used due to their known or suspected “housekeeping” roles, should be avoided due to high expression variability across different tissues or experimental conditions. In this paper we evaluated the stability of 112 genes using three different algorithms: geNorm, NormFinder and RefFinder in spleen samples from BALB/c mice under different experimental conditions (control and *Leishmania infantum*-infected mice). Despite minor discrepancies in the stability ranking shown by the three methods, most genes show very similar performance as RG (either good or poor) across this massive data set. Our results show that some of the genes traditionally used as RG in this model (i.e. *B2m*, *Polr2a* and *Tbp*) are clearly outperformed by others. In particular, the combination of *Il2rg* + *Itgb2* was identified among the best scoring candidate RG for every group of mice and every algorithm used in this experimental model. Finally, we have demonstrated that using “traditional” vs rationally-selected RG for normalization of gene expression data may lead to loss of statistical significance of gene expression changes when using large-scale platforms, and therefore misinterpretation of results. Taken together, our results highlight the need for a comprehensive, high-throughput search for the most stable reference genes in each particular experimental model.

## Introduction

The term leishmaniases includes a spectrum of diseases caused by parasites belonging to genus *Leishmania* that affects millions of people around the world [1]. The global strategies against the disease include improving leishmaniases diagnostic tools and notification as well as providing early treatment for patients, although drug resistance is arising as a major problem. At present, there is no effective vaccine to prevent or treat leishmaniases in humans. A major limitation in vaccine development against leishmaniases is the lack of precise understanding of the particular immunological mechanisms that allow parasite survival in the vertebrate host.

The interaction of the parasite with the host cell induces dramatic changes in transcriptome patterns in the parasite, but also in the target cells [2,3]. The host withstands the infection by inducing innate immune responses, associated with changes of gene expression of numerous genes. Successful parasites can overcome the host´s immune system and colonize the tissues, and this process is deeply dependent on transcriptional reprogramming of numerous genes including those involved in basal cell processes [3]. Analysis of gene expression in infected tissues during leishmaniases will assist with the evaluation of drug and vaccine candidates, and the understanding of the immunological pathways that lead to protection and/or progression of disease.

Real time quantitative PCR (qPCR) is a highly sensitive, specific and accurate technique that allows analysis of gene expression, comparing steady-state mRNA levels between control and test samples [4]. Besides, it can be easily scaled-up in order to perform high-throughput analysis of multiple targets at the same time [5]. Many advances have been made in this field in recent years, such as the development of technical platforms for high-throughput analysis, improving mRNA quantification and internal standard selection [6,7], and the development of powerful software for analysis of qPCR data[8]. One of the drawbacks of qPCR is, however, its sensitivity, that renders the analysis susceptible to small variations in technical protocols and therefore may lead to misinterpretation of results [9,10]. In order to avoid that problem, qPCR results are generally normalized against one or more reference genes (RG) [11] to correct for non-specific experimental variation, such as differences of quantity and quality of mRNA between the samples, enzymatic activity and different transcriptional activity between tissues [12]. These RG need to be genes whose expression remains stable at various conditions across different biological groups. There are a number of studies were the selection of several traditional candidates to RG has been made in mice model using mathematical algorithms [13–20]. All these studies highlight the need to evaluate candidate RG for accurate normalization of gene expression data. The selection of RG candidates in the *Leishmania*-infected mice model has been traditionally focused in genes with known or suspected housekeeping roles: 18S ribosomal RNA [21,22] (*Rn18s*), B-actin [23,24] (*Actb*), Glyceraldehyde-3-phosphate dehydrogenase [25,26] (*Gadph*) and Hypoxanthine-guanine phosphoribosyltransferase [27,28] (*Hprt*). Several authors have evaluated some of them, showing that mRNA expression of genes such as *B2m* [29], *Hprt*, *Pgk1* [30], *Polr2a* [31], *Tbp* [18,19] and *Ubc* [9] are relatively stable, although others like *18SrRNA* and *Actb* [9] should be avoided for normalization due to high variability across different tissues or experimental conditions.

The development of experiments yielding large transcriptome data sets has, jointly with qPCR studies, identified genes differing from the traditional housekeeping genes as most stably transcribed [32–34]. The comprehensive identification of accurate RG for normalization of gene expression data in BALB/c mice, particularly in *Leishmania* target organs like spleen, remains to be completed. In this study we have used three different normalization algorithms for the evaluation of 112 candidate RG genes in BALB/c mice, in order to select the most stable genes for the accurate normalization of gene expression. Our results show that, in this model and using this technical platform, some of the genes traditionally used as RG are clearly outperformed by other genes, and therefore the rational selection of RG should be strongly considered before performing large gene expression analysis.

## Materials and Methods

### Biological Samples

All experiments involving animals were conducted in accordance to both European (2010/63/UE) and Spanish legislation (Law 53/2013), after approval by the Committee for Research Ethics and Animal Welfare (CEIBA) of the University of La Laguna (Permission code: CEIBA2015-0168).

Promastigotes from *Leishmania infantum* (MCAN/ES/98/LLM-274) were grown at 26°C in RPMI medium (Gibco BRL), supplemented with 20% inactivated fetal calf serum (SBFI), 100 ug/ml streptomycin (Sigma-Aldrich, St. Louis, USA) and 100 U/ml of penicillin (Biochrom AG, Berlin, Germany) until reaching stationary phase.

Forty seven BALB/c (purchased to Charles River Laboratories, France) mice (14–15 weeks old) were used in this study. Mice were randomly separated in two groups: (i) 23 control mice and (ii) 24 mice that were infected with 10^6^ stationary-phase *L*. *infantum* promastigotes via tail vein. Mice were euthanized by cervical dislocation and spleens were removed and immediately stored in RNA*later* at -70°C (Sigma-Aldrich, St. Louis, USA). All efforts were made to minimize animal suffering.

### RNA Isolation and Quantification

Total RNA isolation from spleens (9–11 mg) was performed by cell disruption using FastPrep® System (ProScientific, Cedex, France) and Lysing Matrix D (MP Biomedicals, Solon, USA) in TRI-Reagent (Sigma-Aldrich, St. Louis, USA). RNeasy Mini Kit (Qiagen) was subsequently used for mRNA enrichment following manufacturer’s instructions.

Nucleic acid purity was assessed measuring OD_260/280_ and OD_260/230_ ratios using NanoDrop ND-1000 (Thermo). Only samples with OD_260/280_ ratios between 2.10 and 2.2, and OD_260/230_ ratios between 1.8–2.2 were included in this study. RNA integrity number (RIN) was determined using 2100 Bioanalyzer (Agilent Technologies, Santa Clara, United States). RIN was > 7 for all RNA samples included in this study.

### Reverse Transcription and High-Throughput Real-time quantitative PCR (RT-qPCR)

Reverse transcription was performed with random hexamers using the High Capacity cDNA Reverse Transcription Kit (Life Technologies, Carlsbad, CA) according to manufacturer’s instructions. Briefly, 10 μL of total RNA (200 ng/μL) were added to the 2X reverse transcription mix for a total of a 20-μL reaction. Thermal cycler conditions were set to run for 2 h at 37°C, and for 10 min at 75°C followed by a rapid cooling to 4°C. Real-time PCR was performed using QuantStudio 12K Flex Real-Time PCR System (Life Technologies, Carlsbad, CA). Custom TaqMan OpenArray Real-Time PCR Plates were custom-designed, and included 112 Gene Expression Assays organized in 48 subarrays. cDNA samples were loaded into the plates using an OpenArray AccuFill instrument (Applied Biosystems) according to the manufacturer’s protocols. Each sub-array was loaded with 2.5 μl 2X GeneAmp Fast PCR Master Mix (Applied Biosystems), 1 μl 5X TaqMan OpenArray Remix (Applied Biosystems), 0.3 μl RNase-free water, and 1.2 μl cDNA. The thermal cycling protocol was to manufacturer defaults (95°C for 15 seconds, 60°C for 1 minute, for 40 cycles). All reactions were performed in triplicate. QuantStudio 12K Flex Real-Time PCR System (Life Technologies, Carlsbad, CA) calculates Cq values using an algorithm that takes into account the efficiency of each individual curve, called C_rt_ method [35]. The C_rt_ method sets a threshold for each curve individually that is based on the shape of the amplification curve, regardless of the height or variability of the curve in its early baseline fluorescence. The method first estimates a curve that models the reaction efficiency from the amplification curve. It then uses this curve to determine the relative threshold cycle (C_rt_) from the amplification curve, that eliminates the need of “conventional” Real-Time PCR for calculating the efficiency of each reaction. Therefore, Cq values produced by this platform are already corrected for the efficiency of the amplification.

### Assay Design and Selection of Targets

One hundred and twelve genes were included in this study, all of them available as TaqMan Gene Expression Assays (Life Technologies). In order to evaluate their performance, six candidate RG were selected after a literature search of the most commonly used RG for gene expression assays in BALB/c mice: *Hprt*, *Ubc*, *B2m*, *Pkg1*, *Polr2a* and *Tbp*. 106 extra genes were included in the array, all of them involved in different mechanisms of immune system: cytokines, chemokines and their receptors, transcription factors, prostaglandins, costimulatory molecules, enzymes and others. The complete list of genes is shown in S1 Table.

### Normalization Algorithms

Cq values from the 47 samples analyzed were exported for normalization using three different algorithms: geNorm [36], NormFinder [37] and RefFinder [38].

#### geNorm

geNorm normalization was performed in qBase^PLUS^ software (Biogazelle, Gent, Belgium). The geNorm algorithm evaluates RG candidates using two quality parameters: the stability value (M) and the coefficient of variation of the normalized reference gene expression levels. M value is the average pairwise variation of a particular gene with all other control genes included in the analysis. geNorm allows eliminating the worst-scoring candidate RG and recalculating M values for the rest, considering those with lowest M values (M < 0.5) the most stably expressed across the data set. geNorm also determines the number of RG that are required for optimal normalization using V parameter, calculating the pairwise variation (*V*_*n/n+1*_) between the normalization factors of the samples. Generally, V < 0.15 is considered the threshold value below which an additional reference gene is not required for accurate normalization [36]. In brief, the Cq value of every sample, by triplicate, was loaded in qBase^PLUS^ software, with set cut-off values of M < 0.5 and V < 0.15.

#### NormFinder

NormFinder [37] is a Microsoft Excel-based application available online (http://moma.dk/normfinder-software) that identifies the optimal normalization genes among a list of candidates. This tool ranks the candidates genes depending on their expression stability, considering the variations in expression levels between sample subgroups (intergroup variation) as well as within the subgroups (intragroup variation) assigning a stability value for each candidate gene.

#### RefFinder

RefFinder [38] is a website tool available online (http://fulxie.0fees.us/?type=reference) which integrates the four most common normalization algorithms: NormFinder, BestKeeper [39], geNorm and ΔCt [40] method. This tool ranks every candidate RG based on the geometric mean of the four methods mentioned above.

### Normalization of Gene Expression

qBase Plus software was employed to obtain Relative Quantity (RQ) and Normalized Relative Quantity (NRQ) values from the whole data set, following manufacturer’s instructions [41]. Normalization was performed using two different pairs of RG: Il2rg+Itgb2 and Polr2a+Tbp.

### Evaluation of Reference Genes and Statistical Analyses

Gene expression data (NQR) was compared for three different genes (*Cxcl10*, *Ifng* and *Tnf*). Statistical analyses were performed using the non-parametric Mann-Whitney test for pairwise comparison between control and infected groups, using SPSS software, version 20 (IBM). Uncertainty in data accuracy was calculated as described by Remans et al., 2014 [10].

## Results

Spleens from a collection of 47 BALB/c mice, divided in control mice (*n* = 23) and *L*. *infantum* infected-mice (*n* = 24) were analyzed by RT-qPCR in order to determine gene expression of 112 different genes. After determination of the quality and integrity of RNA and cDNA, and in order to validate its performance in RT-qPCR, it was verified that none of our 47 template cDNAs failed to show amplification with all 112 Taqman Assays, indicating that all of them have enough quality to produce at least one RT-PCR result. In fact, the average of positive RT-PCR amplifications was 97 Taqman Assays/sample (out of 112 possible), very high, taking into account QuantStudio 12K Flex Real-Time platform is a semi-automated system performing thousands of reactions at once. Normalization of gene expression in this massive data set (15792 data points: 112 qPCR reactions performed in triplicate using RNA from 47 mice) was performed using geNorm, NormFinder and RefFinder algorithms independently. As a first step, 41 genes had to be excluded from the analysis. The three algorithms used are sensitive to missing data, meaning that if a single Taqman assay did not yield amplification in one mouse, that gene must be excluded for RG identification across the whole data set. Only 1516 qPCR reactions (out of 15792, less than 10%) did not amplify, but that meant that 41 genes could not be included in the analysis to identify the most stable reference genes. Therefore only 71 genes were accepted for the normalization process. The selection of the most stable RG was performed initially using only control (non-infected) mice, in order to evaluate the performance of the candidate RG (the full ranking is included in S2 Table). In a second stage, gene expression stability in *Leishmania*-infected mice group was analyzed (the full ranking is included in S3 Table), as well as using the whole set of mice (the full ranking is included in S4 Table), in order to test the robustness of the evaluation.

### Expression stability of reference gene candidates in control BALB/c mice

**Fig 1:**
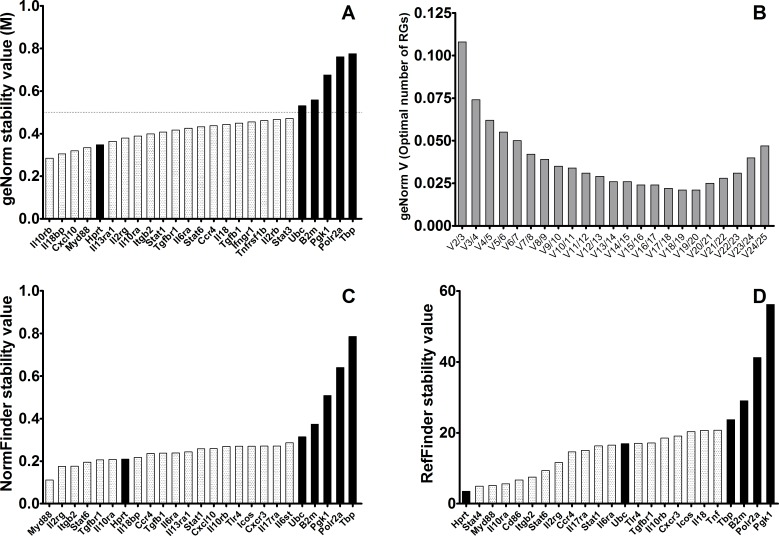
Stability values of the best candidate reference genes (light gray) and 6 six classical reference genes (black bars) in spleen samples of control BALB/c mice. **A)** M-stability value according to geNorm; horizontal line marks the threshold stability value M = 0.5. **B)** Pairwise variation (*V*_*n/n+1*_) between the normalization factors of the samples according to geNorm. **C)** Stability ranking according to NormFinder. **D)** Stability ranking according to RefFinder. Lower values indicate higher stability for all rankings.

Cq data was loaded into qBase^PLUS^ software and geNorm was implemented. According to geNorm algorithm, only *Hprt*, from the typical RG group, showed an M-value below the threshold (M< 0.5), unlike *Ubc*, *B2m*, *Polr2a*, *Pkg1* and *Tbp*, whose M values were higher than 0.5 (Fig 1A). This result indicates that all these typical RG (*Ubc*, *B2m*, *Polr2a*, *Pkg1* and *Tbp*) do not meet the criteria to be used as reference genes in gene expression assays. Moreover, 24 genes related to different immune mechanisms showed better stability than classical RG, reflected as M-values below 0.5 (Fig 1A). geNorm algorithm ranked *Il18bp* and *Il10rb* as the most stable genes (Table 1), enough for optimal normalization according to V parameter (Fig 1B). *NormFinder* analysis of this set of mice ranked *Myd88* and *Il2rg* as the most stably expressed genes (Table 1). Again, the ranking of stability values for the collection of classical RG (Fig 1C), showed that only *Hprt* was ranked among the top-20 genes (7/20), and two of them (*Polr2a* and *Tbp*) were among the 10-worst scoring genes (S2 Table). This same analysis performed with *RefFinder* algorithm ranked *Hprt* and *Stat4* as the most stable candidate reference genes (Table 1). Only *Hprt* and *Ubc* were ranked among the top 20 genes, in first and thirteenth position respectively (Fig 1D).

**Table 1:** Ranking of top-ten reference genes in control BALB/c mice.

Rank	GeNorm	NormFinder	RefFinder
1	Il10rb	**Myd88**	**Hprt**
2	Il18bp	**Il2rg**	Stat4
3	Cxcl10	**Itgb2**	**Myd88**
4	**Myd88**	Stat6	**Il10ra**
5	**Hprt**	Tgfbr1	Cd86
6	Il13ra1	**Il10ra**	**Itgb2**
7	**Il2rg**	**Hprt**	Stat6
8	**Il10ra**	Il18bp	Il2rg
9	**Itgb2**	Ccr4	Ccr4
10	Stat1	Tgfb1	Il17ra

Bold letters indicate those genes that were identical among the top-ten by the three methods. Italics indicate “traditional” reference genes according to literature.

Although the genes selected for normalization for the group of control mice by the three algorithms are different, five of the top 10 genes (*Itgb2*, *Il2rg*, *Il10ra*, *Hprt* and *Myd88*) are identical regardless of the selection algorithm (Table 1), indicating that combinations of these genes are probably the best choice for normalization of gene expression data in experiments using BALB/c mice model.

### Expression Stability of Reference Gene Candidates in BALB/c Mice Infected with *Leishmania infantum*

In order to meet the criteria that mark good reference genes, their expression must remain constant at various biological conditions across different biological groups. For that reason, the same analysis was performed on gene expression data obtained from a group of *Leishmania*-infected mice, and also from the whole set of mice, both control and infected with *L*. *infantum*.

**Fig 2:**
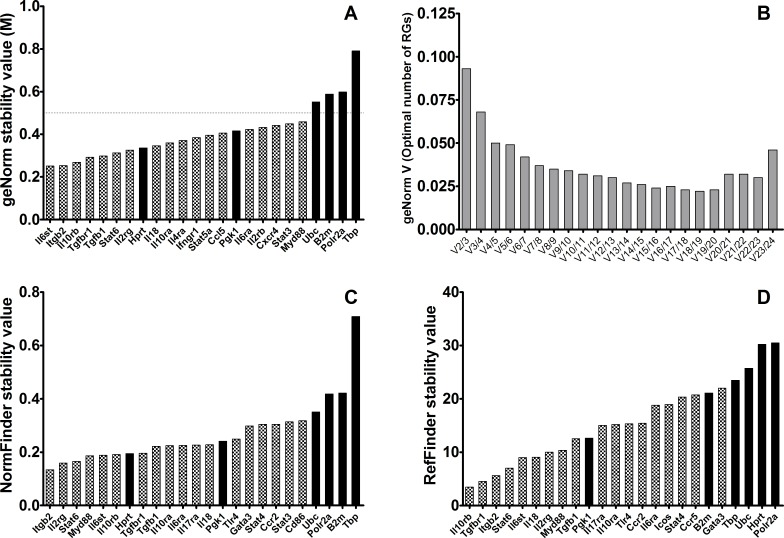
Stability values of the best candidate reference genes (light gray) and 6 six classical reference genes (black bars) in spleen samples of *Leishmania*-infected BALB/c mice. **A)** M-stability value according to geNorm; horizontal line marks the threshold stability value M = 0.5. **B)** Pairwise variation (*V*_*n/n+1*_) between the normalization factors of the samples according to geNorm. **C)** Stability ranking according to NormFinder. **D)** Stability ranking according to RefFinder. Lower values indicate higher stability for all rankings.

geNorm analysis showed that only two commonly used reference genes, *Hprt* and *Pkg1*, show M < 0.5, and therefore are acceptable as RG, ranked 8^th^ and 15^th^ respectively; in contrast, expression stability of *Ubc*, *B2m*, *Polr2a* and *Tbp* are all above the threshold, hence their use is not recommended (Fig 2A). geNorm ranked *Il6st* and *Itgb2* as the most stably expressed genes (Table 2), enough for optimal normalization according to V parameter (Fig 2B). NormFinder analysis also agreed to only rank *Hprt* and *Pkg1* (among the candidate RG) in the top 20 genes, 7^th^ and 14^th^ respectively (Fig 2C), far from the genes whose expression is most stable in the *Leishmania*-infected mice group, *Itgb2* and *Il2rg* (Table 2). In contrast, *Il10rb* and *Tgfbr1* were the top-ranked genes for normalization of gene expression according to *RefFinder* algorithm (Fig 2D). The commonly used reference gene *Pkg1* only ranked 10^th^ in this ranking.

**Table 2:** Ranking of top-ten reference genes in control *Leishmania*-infected BALB/c mice.

Rank	GeNorm	NormFinder	RefFinder
1	**Il6st**	**Itgb2**	**Il10rb**
2	**Itgb2**	**Il2rg**	**Tgfbr1**
3	**Il10rb**	**Stat6**	**Itgb2**
4	**Tgfbr1**	Myd88	**Stat6**
5	**Tgfb1**	**Il6st**	**Il6st**
6	**Stat6**	**Il10rb**	Il18
7	**Il2rg**	*Hprt*	**Il2rg**
8	*Hprt*	**Tgfbr1**	Myd88
9	Il18	**Tgfb1**	**Tgfb1**
10	Il10ra	Il10ra	*Pgk1*

Bold letters indicate those genes that were identical among the top-ten by the three methods. Italics indicate “traditional” reference genes according to literature.

The gene expression of this set of genes is apparently more homogeneous in this group of mice, since 7 of the genes ranked in the top-10 are identical for the three algorithms: *Il2rg*, *Itgb2*, *Stat6*, *Il10rb*, *Tgfbr1*, *Il6st* and *Tgfb1* (Table 2). Interestingly, *Il2rg* and *Itgb2* stand out as two robust reference genes for gene expression analysis in spleens of BALB/c mice, regardless of infection with an intracellular parasite whose target organ is spleen, among others.

The stability of *Il2rg* and *Itgb2* as preferred reference genes in this experimental situation was challenged using a heterogeneous collection of mice, i.e. both healthy control and *Leishmania*-infected mice. The disparity between these two groups must be reflected in the stability values of the genes. Any gene that shows good stability values in “control” group, “infected” and “control+infected” mice will provide a very good reference for accurate normalization of gene expression in this large data set.

**Fig 3:**
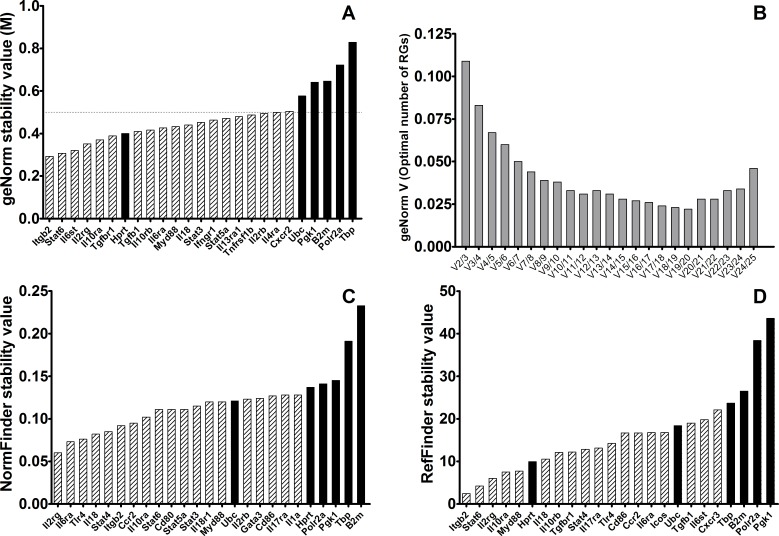
Stability values of the best candidate reference genes (light gray) and 6 six classical reference genes (black bars) in spleen samples of both groups of mice (control and *Leishmania*-infected BALB/c mice). **A)** M-stability value according to geNorm; horizontal line marks the threshold stability value M = 0.5. **B)** Pairwise variation (*V*_*n/n+1*_) between the normalization factors of the samples according to geNorm. **C)** Stability ranking according to NormFinder. **D)** Stability ranking according to RefFinder. Lower values indicate higher stability for all rankings.

geNorm analysis of the whole set of mice revealed that 19 genes showed M < 0.5, only one of them being a traditional RG: *Hprt*, ranked 7/19 (Fig 3A). According to this ranking, *Itgb2* and *Stat6* are the most stable genes (Table 3), and enough for an optimal normalization (Fig 3B). It must be pointed out that *Il2rg* ranks 4^th^ in this analysis. NormFinder analysis revealed *Il2rg* and *Il6ra* as the genes with highest expression stability in this ranking (Table 3). The best scoring classical RG is *Hprt*, as low as 23^rd^ (Fig 3C). RefFinder algorithm was also used to determine the best genes for normalization in the whole data set (Fig 3D). Again, *Hprt* is the only commonly used RG that ranks among the top-20 (6/20). In this ranking, *Itgb2*, *Stat6* and *Il2rg* were most stable genes (Table 3).

**Table 3:** Ranking of top-ten reference genes in control and *Leishmania*-infected BALB/c.mice.

Rank	GeNorm	NormFinder	RefFinder
1	**Itgb2**	**Il2rg**	**Itgb2**
2	**Stat6**	Il6ra	**Stat6**
3	Il6st	Tlr4	**Il2rg**
4	**Il2rg**	Il18	**Il10ra**
5	**Il10ra**	Stat4	Myd88
6	Tgfbr1	**Itgb2**	*Hprt*
7	*Hprt*	Ccr2	Il18
8	Tgfb1	**Il10ra**	Il10rb
9	Il10rb	**Stat6**	Tgfbr1
10	Il6ra	Cd80	Stat4

Bold letters indicate those genes that were identical among the top-ten by the three methods. Italics indicate “traditional” reference genes according to literature.

Four genes, *Il2rg*, *Itgb2*, *Stat6* and *Il10ra*, ranked among the top-10 by the tree algorithms used in the whole set of mice. It is remarkable that *Il2rg* and *Itgb2* are ranked among the best scoring candidate reference genes for every group of mice and every algorithm used, which is indicative of their robustness as reference genes in gene expression analysis in this experimental model, despite its intrinsic heterogeneity.

### Evaluation of *Il2rg* and *Itgb2* as Reference Genes

In order to evaluate the pair of reference genes selected using the three algorithms, *Il2rg* and *Itgb2*, these genes were used to normalize the whole data set, and then compared to the results after normalization with a pair of RG genes chosen upon their extensive use in the literature, *Tbp* [18,19] and *Polr2a* [31]. Hence, normalized gene expression data (NRQ) was compared among control and infected groups for three different genes (*Cxcl10*, *Ifng* and *Tnf*) whose expression is widely analyzed in *Leishmania*-infected BALB/c mice, and are well-known to be overexpressed in this model. These genes code for cytokines that play crucial roles during infection and are overexpressed after *Leishmania* infection [42–45].

**Fig 4:**
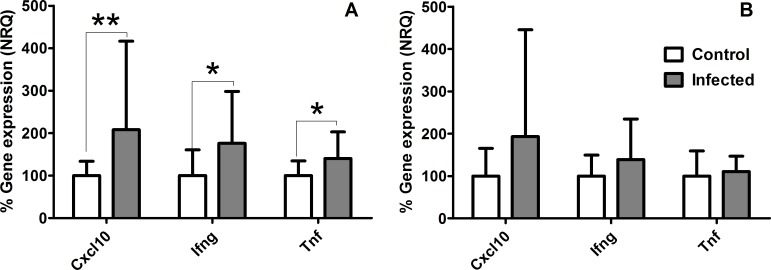
**Relative gene expression of *Cxcl10*, *Ifng* and *Tnf* after normalization with *Igtb2* + *Il2rg* (A) or *Tbp* + *Polr2a* (B).** Mann-Whitney test was used to calculate differences in normalised relative gene expression (NRQ) of *Leishmania*-infected vs control BALB/c mice (expressed as percentage). **p < 0.01; * p < 0.5. The uncertainty values were as follows: Cxcl10, *Igtb2* + *Il2rg* normalization: control mice = 1.03; infected mice = 0.52; *Tbp* + *Polr2a* normalization: control mice = 0.82; infected mice = 0.24. Ifng, *Igtb2* + *Il2rg* normalization: control mice = 0.50; infected mice = -0.09; *Tbp* + *Polr2a* normalization: control mice = 0.38; infected mice = 0.09. Tnf, *Igtb2* + *Il2rg* normalization: control mice = 0.93; infected mice = 0.37; *Tbp* + *Polr2a* normalization: control mice = 0.78; infected mice = 0.46.

Normalization with either set of RG revealed an increased gene expression for *Cxcl10*, *Ifng* and *Tnf* in *Leishmania*–infected mice, as expected upon literature. However, normalization using *Il2rg* and *Itgb2* revealed statistically significant gene expression differences among infected vs control animals for all the genes tested (*Cxcl10* (p < 0.01), *Ifng* and *Tnf* (p < 0.5) after Mann-Whitney tests), whereas normalization with *Tbp* and *Polr2a* did not (Fig 4). This finding emphasizes the relevance of choosing the right set of RG in gene expression experiments in order to produce reliable results.

## Discussion

Identifying the most stable RG for normalization of gene expression analysis under varying experimental conditions has always been a challenge for scientists. During the last few years is becoming increasingly evident that, in order to obtain reliable gene expression data, the adequate selection of RG is key. This problem has been generally addressed by using one or more genes with known or suspected housekeeping roles, but numerous studies are reporting that transcription of these genes can fluctuate considerably under varying experimental conditions and tissues [9,46]. Moreover, most of these papers only test 5–15 candidate RG genes to identify those with the highest stability, overlooking others, not generally described as RG that could outperform the classical RG. In this paper we evaluated the stability of 112 genes using three different algorithms: geNorm, NormFinder and RefFinder in spleen samples from BALB/c mice under different experimental conditions (control and *Leishmania*-infected mice). Despite the discrepancies in the stability ranking shown by the three methods, due to differences in their mathematical approach, most genes show very similar performance as RG (either good or poor) across this massive data set (>15000 qPCR reactions).

Six classical RG were initially selected as candidate RG upon their extensive use as RG for qPCR experiments in the literature: *B2m* [29], *Hprt* [47,48], *Pgk1* [30], *Polr2a* [31], *Tbp* [18,19] and *Ubc* [9]. Surprisingly, classical RG like *B2m*, *Tbp* and *Polr2a* were constantly ranked in our experiments among the less stably expressed genes under these experimental conditions; therefore, we do not recommend the further employment of these genes as normalization controls in RT-qPCR analysis in this model, neither for control nor for *Leishmania*-infected BALB/c mice.

Only *Hprt* was ranked among the 10 most stable genes for most of our analyses, both in control and infected mice, and consequently is the only classical RG that we would consider using in future experiments. This finding is in agreement with several papers [49–52] that evaluated classical RG candidates in mouse intestine, adipocytes, hepatic steatosis and brain respectively. On the contrary, in other studies in mice muscle [53] and non-B lymphocytes [9] the use of *Hprt* gene as RG was not recommended due to considerably variation of gene expression. These discrepancies only highlight the need for a comprehensive screening of adequate RG for each experimental design in order to obtain reliable results.

Our search for the best RG in spleen samples of BALB/c mice revealed that *Il18bp* + *Il10rb* was the best combination of genes according to geNorm, *Myd88* + *Il2rg* for *NormFinder* and *Hprt* + *Stat4* for *RefFinder*. Despite this apparent disagreement, five of 10 the top-ranked genes are identical regardless of the algorithm: *Itgb2*, *Il2rg*, *Il10ra*, *Hprt* and *Myd88*. The agreement between the algorithms is even higher when analyzing *Leishmania*-infected BALB/c mice: seven out of the 10 the top-ranked genes are the same: *Il2rg*, *Itgb2*, *Stat6*, *Il10rb*, *Tgfbr1*, *Il6st* and *Tgfb1*; being *Il6st*+*Itgb2* the best combination for geNorm, *Itgb2*+*Il2rg* for NormFinder and *Il10rb*+*Tgfbr1* for RefFinder.

Given the interest in transcriptome changes during *Leishmania* infection in spleens of BALB/c mice, finally both groups (control and infected) were taken together to identify the most stably expressed genes. geNorm ranked *Itgb2*+*Stat6* as the best combination of RG, NormFinder chose *Il2rg*+*Il6ra* and RefFinder proposed *Itgb2*+*Stat6*. Four of the best 10 RG proposed by the three algorithms are the same: *Itgb2*, *Stat6*, *Il2rg* e *Il10ra*.

It is remarkable that, despite the natural heterogeneity of this model, *Il2rg* and *Itgb2* are always ranked among the best scoring candidate reference genes for every group of mice (control/*Leishmania*-infected/altogether) and every algorithm used, which is indicative of their robustness as reference genes in gene expression analysis using a high-throughput platform in this experimental model. It may seem surprising, given that most of these genes are related to immune response and have never been described as RG in the literature. *Il2rg* encodes a transmembrane protein that is a common subunit of several interleukin receptor complexes. *Itgb2* encodes an integrin beta chain, which combines with multiple different alpha chains to form different integrin heterodimers, cell-surface proteins that participate in cell adhesion as well as cell-surface mediated signaling. Although not “traditional” housekeeping genes, both have roles related to homeostasis of immune system. After the analysis of this extensive collection of data, it is clear that these genes present higher expression stability in this model than the commonly used RG, and should be considered a suitable alternative. A similar finding was described by Müller et al., (2015) [54] in a very different model, pathogen-infected tomato leaves. After a genome-wide microarray analysis and RT-PCR, they identified a set of very stably expressed genes that were never described for that purpose before, and were always evaluated as more stable than the traditional housekeeping genes included for comparison.

Finally, in order to demonstrate the effects of using “traditional” vs rationally-selected RG for normalization of gene expression data in our model, changes in expression of *Cxcl10*, *Ifng* and *Tnf* genes in control/*Leishmania*-infected mice was determined after normalization using the pair of RG identified by our screening, *Il2rg* + *Itgb2*, or two classical RG, *Tbp* +*Polr2a*. *Cxcl10*, *Ifng* and *Tnf* gene expression is widely described to increase during leishmaniases in BALB/c mice [42–45]. After initial *Leishmania* infection of macrophages and dendritic cells, these cells produce proinflamatory cytokines and chemokines, such as TNF-α and CXCL-10, that in turn recruit new host cells (natural killer cells (NK), monocytes and neutrophils) to the infection site [55,56]. Similarly, NK cells produce IFN-γ in an attempt to activate macrophages to phagocityze parasites. Normalization with *Tbp*+*Polr2a* failed to reveal statistically significant gene expression differences among infected vs control animals for all the genes tested. In contrast, normalization using *Il2rg* + *Itgb2* showed differential gene expression for *Cxcl10*, *Ifng* and *Tnf* as expected.

Taken together, our results highlight the need for a comprehensive search of the most stable reference genes in each particular experimental model. The use of “traditional” or non-evaluated reference genes in gene expression experiments may drive scientists to extract flawed conclusions from their research, which is particularly relevant when using high-throughput platforms like QuantStudio 12K Flex Real-Time PCR System. In our model of *Leishmania* infected BALB/c mice, extensively used for preliminary testing of vaccine candidates against leishmaniases (reviewed in de Oliveira et al., 2009 and references therein) [57], classical RG like *B2m*, *Tbp* and *Polr2a* have shown very poor stability as RGs in this large-scale gene-expression platform, and therefore we cannot recommend their use as normalization controls. In addition, our results demonstrate the advantage of a comprehensive, high-throughput approach to identify suitable reference genes for transcriptomic studies. In this particular experimental model (spleen samples of *Leishmania* infected BALB/c mice) our approach has identified and evaluated *Il2rg* + *Itgb2* as the best combination of RG, but it is unclear whether this results can be extrapolated to other samples or animal models. Therefore, scientists planning large qPCR gene-expression experiments should make all efforts to identify the best RGs for their particular experimental conditions using a comprehensive high-throughput approach. Besides, validation of the selected RGs in each and every experimental approach is another fundamental issue that should be addressed in order to obtain reliable results from these high-throughput experimental designs.

The data discussed in this publication have been deposited in NCBI’s Gene Expression Omnibus [58] and are accessible through GEO Series accession number GSE80709 (https://www.ncbi.nlm.nih.gov/geo/query/acc.cgi?acc=GSE80709).

## Supporting Information

S1 Table List of 112 TaqMan assays used for RT-qPCR analysis using QuantStudio™ 12K Flex Real-Time PCR System. (DOCX)

S2 Table Stability values of all 71 candidate reference genes for spleen samples of control BALB/c mice, ranked by geNorm, NormFinder and RefFinder. (DOCX)

S3 Table Stability values of all 71 candidate reference genes for spleen samples of *Leishmania*-infected BALB/c mice, ranked by geNorm, NormFinder and RefFinder. (DOCX)

S4 Table Stability values of all 71 candidate reference genes for spleen samples of control and *Leishmania*-infected BALB/c mice, ranked by geNorm, NormFinder and RefFinder. (DOCX)

## References

[1] World Health Organization. Control of the leishmaniases. World Health Organ Tech Rep Ser. 2010;949: xii–xiii, 1–186, back cover. Available: http://www.ncbi.nlm.nih.gov/pubmed/21485694 21485694

[2] Katara G, Raj A, Kumar R, Avishek K, Kaushal H, Ansari N, et al Analysis of localized immune responses reveals presence of Th17 and Treg cells in cutaneous leishmaniases due to Leishmania tropica. BMC Immunol. 2013;14: 52 10.1186/1471-2172-14-52 24267152 PMC3840658

[3] Ovalle-Bracho C, Franco-Muñoz C, Londoño-Barbosa D, Restrepo-Montoya D, Clavijo-Ramírez C. Changes in Macrophage Gene Expression Associated with Leishmania (Viannia) braziliensis Infection. PLOS ONE. 2015;10: e0128934 10.1371/journal.pone.0128934 26052705 PMC4460072

[4] VanGuilder HD, Vrana KE, Freeman WM. Twenty-five years of quantitative PCR for gene expression analysis. Biotechniques. 2008;44: 619–26. 10.2144/000112776 18474036

[5] Heid CA, Stevens J, Livak KJ, Williams PM. Real time quantitative PCR. Genome Res. 1996;6: 986–94. Available: http://www.ncbi.nlm.nih.gov/pubmed/8908518 8908518 10.1101/gr.6.10.986

[6] Thellin O, ElMoualij B, Heinen E, Zorzi W. A decade of improvements in quantification of gene expression and internal standard selection. Biotechnol Adv. 2009;27: 323–333. 10.1016/j.biotechadv.2009.01.010 19472509

[7] Rebouças EDL, Jackson J, Passos MJ, Renato J, Passos DS, Hurk R Van Den, et al Real Time PCR and Importance of Housekeepings Genes for Normalization and Quantification of mRNA Expression in Different Tissues. Brazilian Arch Biol Technol. 2013;56: 143–154.

[8] Pabinger S, Rödiger S, Kriegner A, Vierlinger K, Weinhäusel A. A survey of tools for the analysis of quantitative PCR (qPCR) data. Biomol Detect Quantif. 2014;1: 23–33. 10.1016/j.bdq.2014.08.002 27920994 PMC5129434

[9] Albershardt TC, Iritani BM, Ruddell A. Evaluation of reference genes for quantitative PCR analysis of mouse lymphocytes. J Immunol Methods. 2012;384: 196–9. 10.1016/j.jim.2012.07.020 22884776 PMC3432750

[10] Remans T, Keunen E, Bex GJ, Smeets K, Vangronsveld J, Cuypers A. Reliable gene expression analysis by reverse transcription-quantitative PCR: reporting and minimizing the uncertainty in data accuracy. Plant Cell. 2014;26: 3829–37. 10.1105/tpc.114.130641 25361954 PMC4247583

[11] Thellin O, Zorzi W, Lakaye B, De Borman B, Coumans B, Hennen G, et al Housekeeping genes as internal standards: use and limits. J Biotechnol. 1999;75: 291–5. Available: http://www.ncbi.nlm.nih.gov/pubmed/10617337 10617337 10.1016/s0168-1656(99)00163-7

[12] Bustin SA, Benes V, Garson JA, Hellemans J, Huggett J, Kubista M, et al The MIQE guidelines: minimum information for publication of quantitative real-time PCR experiments. Clin Chem. 2009;55: 611–22. 10.1373/clinchem.2008.112797 19246619

[13] Frericks M, Esser C. A toolbox of novel murine house-keeping genes identified by meta-analysis of large scale gene expression profiles. Biochim Biophys Acta. 2008;1779: 830–7. 10.1016/j.bbagrm.2008.08.007 18790095

[14] Jeong J-KK, Kang M-HH, Gurunathan S, Cho S-GG, Park C, Seo HG, et al Evaluation of reference genes in mouse preimplantation embryos for gene expression studies using real-time quantitative RT-PCR (RT-qPCR). BMC Res Notes. 2014;7: 675 10.1186/1756-0500-7-675 25256308 PMC4181407

[15] Thomas KC, Zheng XF, Garces Suarez F, Raftery JM, Quinlan KGR, Yang N, et al Evidence based selection of commonly used RT-qPCR reference genes for the analysis of mouse skeletal muscle. PLOS ONE. 2014;9: e88653 10.1371/journal.pone.0088653 24523926 PMC3921188

[16] Gong ZK, Wang SJ, Huang YQ, Zhao RQ, Zhu QF, Lin WZ. Identification and validation of suitable reference genes for RT-qPCR analysis in mouse testis development. Mol Genet genomics. 2014;289: 1157–1169. 10.1007/s00438-014-0877-6 24952483

[17] Arsenijevic T, Grégoire F, Delforge V, Delporte C, Perret J. Murine 3T3-L1 adipocyte cell differentiation model: validated reference genes for qPCR gene expression analysis. PLOS ONE. 2012;7: e37517 10.1371/journal.pone.0037517 22629413 PMC3358259

[18] Wang F, Wang J, Liu D, Su Y. Normalizing genes for real-time polymerase chain reaction in epithelial and nonepithelial cells of mouse small intestine. Anal Biochem. 2010;399: 211–7. 10.1016/j.ab.2009.12.029 20036209

[19] Tatsumi K, Ohashi K, Taminishi S, Okano T, Yoshioka A, Shima M. Reference gene selection for real-time RT-PCR in regenerating mouse livers. Biochem Biophys Res Commun. 2008;374: 106–10. 10.1016/j.bbrc.2008.06.103 18602371

[20] Yin R, Tian F, Frankenberger B, de Angelis MH, Stoeger T. Selection and evaluation of stable housekeeping genes for gene expression normalization in carbon nanoparticle-induced acute pulmonary inflammation in mice. Biochem Biophys Res Commun. 2010;399: 531–6. 10.1016/j.bbrc.2010.07.104 20678479

[21] Teixeira C, Gomes R, Oliveira F, Meneses C, Gilmore DC, Elnaiem DEA, et al Characterization of the early inflammatory infiltrate at the feeding site of infected sand flies in mice protected from vector-transmitted Leishmania major by exposure to uninfected bites. PLoS Negl Trop Dis. 2014;8: e2781 10.1371/journal.pntd.0002781 24762408 PMC3998922

[22] Osorio EY, Zhao W, Espitia C, Saldarriaga O, Hawel L, Byus C V, et al Progressive visceral leishmaniases is driven by dominant parasite-induced STAT6 activation and STAT6-dependent host arginase 1 expression. PLoS Pathog. 2012;8: e1002417 10.1371/journal.ppat.1002417 22275864 PMC3261917

[23] Paun A, Bankoti R, Joshi T, Pitha PM, Stäger S. Critical role of IRF-5 in the development of T helper 1 responses to Leishmania donovani infection. PLoS Pathog. 2011;7: e1001246 10.1371/journal.ppat.1001246 21253574 PMC3017120

[24] Singh AK, Mukhopadhyay C, Biswas S, Singh VK, Mukhopadhyay CK. Intracellular pathogen Leishmania donovani activates hypoxia inducible factor-1 by dual mechanism for survival advantage within macrophage. PLOS ONE. 2012;7: e38489 10.1371/journal.pone.0038489 22701652 PMC3373497

[25] Bhattacharjee S, Bhattacharjee A, Majumder S, Majumdar SB, Majumdar S. Glycyrrhizic acid suppresses Cox-2-mediated anti-inflammatory responses during Leishmania donovani infection. J Antimicrob Chemother. 2012;67: 1905–14. 10.1093/jac/dks159 22589456

[26] Barreto-de-Souza V, Ferreira PLC, de Carvalho Vivarini A, Calegari-Silva T, Soares DC, Regis EG, et al IL-27 enhances Leishmania amazonensis infection via ds-RNA dependent kinase (PKR) and IL-10 signaling. Immunobiology. 2014; 1–8. 25466588 10.1016/j.imbio.2014.11.006

[27] Rodrigues OR, Marques C, Soares-Clemente M, Ferronha MH, Santos-Gomes GM. Identification of regulatory T cells during experimental Leishmania infantum infection. Immunobiology. 2009;214: 101–11. 10.1016/j.imbio.2008.07.001 19167988

[28] Phillips R, Svensson M, Aziz N, Maroof A, Brown N, Beattie L, et al Innate killing of Leishmania donovani by macrophages of the splenic marginal zone requires IRF-7. PLoS Pathog. 2010;6: e1000813 10.1371/journal.ppat.1000813 20300600 PMC2837405

[29] Stephens AS, Stephens SR, Morrison NA. Internal control genes for quantitative RT-PCR expression analysis in mouse osteoblasts, osteoclasts and macrophages. BMC Res Notes. 2011;4: 410 10.1186/1756-0500-4-410 21996334 PMC3204251

[30] Veazey KJ, Golding MC. Selection of stable reference genes for quantitative rt-PCR comparisons of mouse embryonic and extra-embryonic stem cells. PLOS ONE. 2011;6: e27592 10.1371/journal.pone.0027592 22102912 PMC3213153

[31] Brattelid T, Winer LH, Levy FO, Liestøl K, Sejersted OM, Andersson KB. Reference gene alternatives to Gapdh in rodent and human heart failure gene expression studies. BMC Mol Biol. 2010;11: 22 10.1186/1471-2199-11-22 20331858 PMC2907514

[32] Matarin M, Salih DA, Yasvoina M, Cummings DM, Guelfi S, Liu W, et al A genome-wide gene-expression analysis and database in transgenic mice during development of amyloid or tau pathology. Cell Rep. 2015;10: 633–44. 10.1016/j.celrep.2014.12.041 25620700

[33] Narsai R, Ivanova A, Ng S, Whelan J. Defining reference genes in Oryza sativa using organ, development, biotic and abiotic transcriptome datasets. BMC Plant Biol. 2010;10: 56 10.1186/1471-2229-10-56 20353606 PMC2923530

[34] Czechowski T, Stitt M, Altmann T, Udvardi MK, Scheible W-R. Genome-wide identification and testing of superior reference genes for transcript normalization in Arabidopsis. Plant Physiol. 2005;139: 5–17. 10.1104/pp.105.063743 16166256 PMC1203353

[35] Crt, a relative threshold method for qPCR data analysis on the QuantStudioTM 12K Flex system with OpenArray® technology. Appl Biosyst QuantStudioTM 12K Flex Real-Time PCR Syst Appl Note. 2014;CO28730: 4.

[36] Vandesompele J, Preter K De, Poppe B, Roy N Van, Paepe A De, De Preter K, et al Accurate normalization of real-time quantitative RT-PCR data by geometric averaging of multiple internal control genes. Genome Biol. 2002;3: 1–12. Available: http://www.pubmedcentral.nih.gov/articlerender.fcgi?artid=126239&tool=pmcentrez&rendertype=abstract 10.1186/gb-2002-3-7-research0034 PMC126239 12184808

[37] Andersen CL, Jensen JL, Ørntoft TF. Normalization of Real-Time Quantitative Reverse Transcription-PCR Data: A Model-Based Variance Estimation Approach to Identify Genes Suited for Normalization, Applied to Bladder and Colon Cancer Data Sets Normalization of Real-Time Quantitative Reverse. Cancer Res. 2004;64: 5245–5250. 15289330 10.1158/0008-5472.CAN-04-0496

[38] Xie F, Xiao P, Chen D, Xu L, Zhang B. miRDeepFinder: a miRNA analysis tool for deep sequencing of plant small RNAs. Plant Mol Biol. 2012; 10.1007/s11103-012-9885-2 22290409

[39] Pfaffl MW, Tichopad A, Prgomet C, Neuvians TP. Determination of stable housekeeping genes, differentially regulated target genes and sample integrity: BestKeeper–Excel-based tool using pair-wise correlations. Biotechnol Lett. 2004;26: 509–515. 10.1023/B:BILE.0000019559.84305.47 15127793

[40] Silver N, Best S, Jiang J, Thein SL. Selection of housekeeping genes for gene expression studies in human reticulocytes using real-time PCR. BMC Mol Biol. 2006;7: 33 10.1186/1471-2199-7-33 17026756 PMC1609175

[41] Hellemans J et al, Mortier G, De Paepe A, Speleman F, Vandesompele J. qBase relative quantification framework and software for management and automated analysis of real-time quantitative PCR data. Genome Biol. 2007;8: R19 10.1186/gb-2007-8-2-r19 17291332 PMC1852402

[42] Strauss-Ayali D, Baneth G, Jaffe CL. Splenic immune responses during canine visceral leishmaniases. Vet Res. 2007;38: 547–64. 10.1051/vetres:2007015 17540157

[43] Nascimento MSL, Albuquerque TD, Do-Valle-Matta MA, Caldas IS, Diniz LF, Talvani A, et al Naturally Leishmania infantum-infected dogs display an overall impairment of chemokine and chemokine receptor expression during visceral leishmaniases. Vet Immunol Immunopathol. 2013;153: 202–8. 10.1016/j.vetimm.2013.02.015 23545087

[44] Cezário GA, de Oliveira LR, Peresi E, Nicolete VC, Polettini J, de Lima CR, et al Analysis of the expression of toll-like receptors 2 and 4 and cytokine production during experimental Leishmania chagasi infection. Mem Inst Oswaldo Cruz. 2011;106: 573–583. 10.1590/S0074-02762011000500010 21894379

[45] Faleiro RJ, Kumar R, Hafner LM, Engwerda CR. Immune regulation during chronic visceral leishmaniases. PLoS Negl Trop Dis. 2014;8: e2914 10.1371/journal.pntd.0002914 25010815 PMC4091888

[46] Gutierrez L, Mauriat M, Pelloux J, Bellini C, Van Wuytswinkel O. Towards a Systematic Validation of References in Real-Time RT-PCR. Plant Cell. 2008;20: 1734–5. 10.1105/tpc.108.059774 18664615 PMC2518241

[47] Hurdayal R, Nieuwenhuizen NE, Revaz-Breton M, Smith L, Hoving JC, Parihar SP, et al Deletion of IL-4 receptor alpha on dendritic cells renders BALB/c mice hypersusceptible to Leishmania major infection. PLoS Pathog. 2013;9: e1003699 10.1371/journal.ppat.1003699 24204259 PMC3812013

[48] Weinkopff T, de Oliveira CI, de Carvalho AM, Hauyon-La Torre Y, Muniz AC, Miranda JC, et al Repeated exposure to Lutzomyia intermedia sand fly saliva induces local expression of interferon-inducible genes both at the site of injection in mice and in human blood. PLOS Negl Trop Dis. 2014;8: e2627 10.1371/journal.pntd.0002627 24421912 PMC3888461

[49] Zhang J, Tang H, Zhang Y, Deng R, Shao L, Liu Y, et al Identification of suitable reference genes for quantitative RT-PCR during 3T3-L1 adipocyte differentiation. Int J Mol Med. 2014;33: 1209–18. 10.3892/ijmm.2014.1695 24626784

[50] Xu L, Ma X, Cui B, Li X, Ning G, Wang S. Selection of reference genes for qRT-PCR in high fat diet-induced hepatic steatosis mice model. Mol Biotechnol. 2011;48: 255–62. 10.1007/s12033-010-9366-2 21184202

[51] Timaru-Kast R, Herbig EL, Luh C, Engelhard K, Thal SC. Influence of Age on Cerebral Housekeeping Gene Expression for Normalization of Quantitative Polymerase Chain Reaction after Acute Brain Injury in Mice. J Neurotrauma. 2015;32: 1777–88. 10.1089/neu.2014.3784 26102571

[52] Li X, Qiao J, Yang N, Mi H, Chu P, Xia Y, et al Identification of Suitable Reference Genes for Normalization of Real-Time Quantitative Polymerase Chain Reaction in an Intestinal Graft-Versus-Host Disease Mouse Model. Transplant Proc. 2015;47: 2017–25. 10.1016/j.transproceed.2015.06.017 26293091

[53] Nakao R, Yamamoto S, Yasumoto Y, Kadota K, Oishi K. Impact of denervation-induced muscle atrophy on housekeeping gene expression in mice. Muscle Nerve. 2015;51: 276–281. 10.1002/mus.24310 24910410

[54] Müller OA, Grau J, Thieme S, Prochaska H, Adlung N, Sorgatz A, et al Genome-Wide Identification and Validation of Reference Genes in Infected Tomato Leaves for Quantitative RT-PCR Analyses. PLOS ONE. 2015;10: e0136499 10.1371/journal.pone.0136499 26313760 PMC4552032

[55] Stanley AC, Engwerda CR. Balancing immunity and pathology in visceral leishmaniases. Immunol Cell Biol. 2007;85: 138–47. 10.1038/sj.icb7100011 17146466

[56] Liaskou E, Wilson D V, Oo YH. Innate immune cells in liver inflammation. Mediators Inflamm. 2012;2012: 949157 10.1155/2012/949157 22933833 PMC3425885

[57] de Oliveira CI, Nascimento IP, Barral A, Soto M, Barral-Netto M. Challenges and perspectives in vaccination against leishmaniases. Parasitol Int. 2009;58: 319–24. 10.1016/j.parint.2009.07.013 19698801

[58] Edgar R, Domrachev M, Lash AE. Gene Expression Omnibus: NCBI gene expression and hybridization array data repository. Nucleic Acids Res. 2002;30: 207–10. Available: http://www.pubmedcentral.nih.gov/articlerender.fcgi?artid=99122&tool=pmcentrez&rendertype=abstract 11752295 10.1093/nar/30.1.207 PMC99122

