# Supplementary material for: The Challenge of Stability in High-Throughput Gene Expression Analysis: Comprehensive Selection and Evaluation of Reference Genes for BALB/c Mice Spleen Samples in the Leishmania infantum Infection Model
Source: PLoS One. 2016 Sep 26;11(9):e0163219. doi: 10.1371/journal.pone.0163219 (PMC5036817; doi:10.1371/journal.pone.0163219)
Supplement: S1 Table — (DOCX) [file pone.0163219.s001.docx]

**S1 Table. List of 112 TaqMan assays used for RT-qPCR analysis using QuantStudio™ 12K Flex Real-Time PCR System.**

| Nº | Gene Symbol | Assay ID | Gene Name |
| --- | --- | --- | --- |
| **1** | **Arg1** | **Mm00475988_m1** | **Arginase, liver** |
| 2 | *B2m* | Mm00437762_m1 | Beta-2 microglobulin |
| **3** | **Ccl17** | **Mm00516136_m1** | **Chemokine (C-C motif) ligand 17** |
| **4** | **Ccl2** | **Mm00441242_m1** | **Chemokine (C-C motif) ligand 2** |
| 5 | Ccl22 | Mm00436439_m1 | Chemokine (C-C motif) ligand 22 |
| **6** | **Ccl3** | **Mm00441258_m1** | **Chemokine (C-C motif) ligand 3** |
| **7** | **Ccl4** | **Mm00443111_m1** | **Chemokine (C-C motif) ligand 4** |
| 8 | Ccl5 | Mm01302427_m1 | Chemokine (C-C motif) ligand 5 |
| **9** | **Ccl7** | **Mm00443113_m1** | **Chemokine (C-C motif) ligand 7** |
| 10 | Ccr1 | Mm01216147_m1 | Chemokine (C-C motif) receptor 1 |
| 11 | Ccr2 | Mm01216173_m1 | Chemokine (C-C motif) receptor 2 |
| 12 | Ccr4 | Mm00438271_m1 | Chemokine (C-C motif) receptor 4 |
| 13 | Ccr5 | Mm01216171_m1 | Chemokine (C-C motif) receptor 5 |
| 14 | Ccr7 | Mm01301785_m1 | Chemokine (C-C motif) receptor 7 |
| **15** | **Cd28** | **Mm00483137_m1** | **CD28 antigen** |
| **16** | **Cd40** | **Mm00441891_m1** | **CD40 antigen** |
| **17** | **Cd40lg** | **Mm00441911_m1** | **CD40 ligand** |
| 18 | Cd54 | Mm00516023_m1 | Intercellular adhesion molecule 1 |
| 19 | Cd80 | Mm00711660_m1 | CD80 antigen |
| 20 | Cd86 | Mm00444543_m1 | CD86 antigen |
| **21** | **Ctla4** | **Mm00486849_m1** | **Cytotoxic T-lymphocyte-associated protein 4** |
| **22** | **Cxcl1** | **Mm04207460_m1** | **Chemokine (C-X-C motif) ligand 1** |
| 23 | Cxcl10 | Mm00445235_m1 | Chemokine (C-X-C motif) ligand 10 |
| **24** | **Cxcl2** | **Mm00436450_m1** | **Chemokine (C-X-C motif) ligand 2** |
| 25 | Cxcl9 | Mm00434946_m1 | Chemokine (C-X-C motif) ligand 9 |
| 26 | Cxcr2 | Mm00438258_m1 | Chemokine (C-X-C motif) receptor 2 |
| 27 | Cxcr3 | Mm00438259_m1 | Chemokine (C-X-C motif) receptor 3 |
| 28 | Cxcr4 | Mm99999055_m1 | Chemokine (C-X-C motif) receptor 4 |
| **29** | **Ebi3** | **Mm00469294_m1** | **Epstein-Barr virus induced gene 3** |
| **30** | **Foxp3** | **Mm00475162_m1** | **Forkhead box P3** |
| 31 | Gata3 | Mm00484683_m1 | GATA binding protein 3 |
| 32 | *Hprt* | Mm00446968_m1 | Hypoxanthine guanine phosphoribosyl transferase |
| 33 | Icam2 | Mm00494862_m1 | Iintercellular adhesion molecule 2 |
| 34 | Icos | Mm00497600_m1 | Inducible T cell co-stimulator |
| 35 | Icosl | Mm00497237_m1 | Icos ligand |
| **36** | **Ifng** | **Mm01168134_m1** | **Interferon gamma** |
| 37 | Ifngr1 | Mm00599890_m1 | Interferon gamma receptor 1 |
| 38 | Ifngr2 | Mm00492626_m1 | Interferon gamma receptor 2 |
| **39** | **Il10** | **Mm00439614_m1** | **Interleukin 10** |
| 40 | Il10ra | Mm00434151_m1 | Interleukin 10 receptor, alpha |
| 41 | Il10rb | Mm00434157_m1 | Interleukin 10 receptor, beta |
| 42 | Il12a | Mm00434165_m1 | Interleukin 12a |
| **43** | **Il12b** | **Mm00434174_m1** | **Interleukin 12b** |
| **44** | **Il12rb1** | **Mm00434189_m1** | **Interleukin 12 receptor, beta 1** |
| 45 | Il12rb2 | Mm00434200_m1 | Interleukin 12 receptor, beta 2 |
| **46** | **Il13** | **Mm00434204_m1** | **Interleukin 13** |
| 47 | Il13ra1 | Mm00446726_m1 | Interleukin 13 receptor, alpha 1 |
| **48** | **Il13ra2** | **Mm00515166_m1** | **Interleukin 13 receptor, alpha 2** |
| **49** | **Il17a** | **Mm00439618_m1** | **Interleukin 17A** |
| **50** | **Il17f** | **Mm00521423_m1** | **Interleukin 17F** |
| 51 | Il17ra | Mm00434214_m1 | Interleukin 17 receptor A |
| 52 | Il18 | Mm00434225_m1 | Interleukin 18 |
| 53 | Il18bp | Mm00456733_m1 | Interleukin 18 binding protein |
| 54 | Il18r1 | Mm00515178_m1 | Interleukin 18 receptor 1 |
| **55** | **Il18rap** | **Mm00516053_m1** | **Interleukin 18 receptor accessory protein** |
| 56 | Il1a | Mm00439620_m1 | Interleukin 1 alpha |
| 57 | Il1b | Mm00434228_m1 | Interleukin 1 beta |
| 58 | Il1r1 | Mm00434237_m1 | Interleukin 1 receptor, type I |
| **59** | **Il1rap** | **Mm00492638_m1** | **Interleukin 1 receptor accessory protein** |
| 60 | Il1rn | Mm00446186_m1 | Interleukin 1 receptor antagonist |
| **61** | **Il2** | **Mm00434256_m1** | **Interleukin 2** |
| **62** | **Il21** | **Mm00517640_m1** | **Interleukin 21** |
| 63 | Il21r | Mm00600319_m1 | Interleukin 21 receptor |
| **64** | **Il22** | **Mm00444241_m1** | **Interleukin 22** |
| **65** | **Il22ra1** | **Mm01192943_m1** | **Interleukin 22 receptor, alpha 1** |
| **66** | **Il22ra2** | **Mm01192969_m1** | **Interleukin 22 receptor, alpha 2** |
| **67** | **Il23a** | **Mm00518984_m1** | **Interleukin 23, alpha subunit p19** |
| **68** | **Il23r** | **Mm00519943_m1** | **Interleukin 23 receptor** |
| **69** | **Il27** | **Mm00461162_m1** | **Interleukin 27** |
| 70 | Il27ra | Mm00497259_m1 | Interleukin 27 receptor, alpha |
| 71 | Il2ra | Mm01340213_m1 | Interleukin 2 receptor, alpha chain |
| 72 | Il2rb | Mm00434268_m1 | Interleukin 2 receptor, beta chain |
| 73 | Il2rg | Mm00442885_m1 | Interleukin 2 receptor, gamma chain |
| **74** | **Il4** | **Mm00445259_m1** | **Interleukin 4** |
| 75 | Il4ra | Mm01275139_m1 | Interleukin 4 receptor, alpha |
| **76** | **Il5** | **Mm00439646_m1** | **Interleukin 5** |
| 77 | Il5ra | Mm00434284_m1 | Interleukin 5 receptor, alpha |
| **78** | **Il6** | **Mm00446190_m1** | **Interleukin 6** |
| 79 | Il6ra | Mm00439653_m1 | Interleukin 6 receptor, alpha |
| 80 | Il6st | Mm00439665_m1 | Interleukin 6 signal transducer |
| 81 | Itgal | Mm00801807_m1 | Integrin alpha L |
| **82** | **Itgam** | **Mm00434455_m1** | **Integrin alpha M** |
| 83 | Itgb2 | Mm00434513_m1 | Integrin beta 2 |
| 84 | Myd88 | Mm00440338_m1 | Myeloid differentiation primary response gene 88 |
| **85** | **Nos2** | **Mm00440502_m1** | **Nitric oxide synthase 2, inducible** |
| **86** | **Nox4** | **Mm00479246_m1** | **NADPH oxidase 4** |
| 87 | *Pgk1* | Mm00435617_m1 | Phosphoglycerate kinase 1 |
| 88 | *Polr2a* | Mm00839493_m1 | Polymerase (RNA) II (DNA directed) polypeptide A |
| 89 | Ptges | Mm00452105_m1 | Prostaglandin E synthase |
| **90** | **Ptgs2** | **Mm00478374_m1** | **Prostaglandin-endoperoxide synthase 2** |
| 91 | Stat1 | Mm00439531_m1 | Signal transducer and activator of transcription 1 |
| 92 | Stat3 | Mm01219775_m1 | Signal transducer and activator of transcription 3 |
| 93 | Stat4 | Mm00448890_m1 | Signal transducer and activator of transcription 4 |
| 94 | Stat5a | Mm00839861_m1 | Signal transducer and activator of transcription 5A |
| 95 | Stat5b | Mm00839889_m1 | Signal transducer and activator of transcription 5B |
| 96 | Stat6 | Mm01160477_m1 | Signal transducer and activator of transcription 6 |
| 97 | *Tbp* | Mm00446971_m1 | TATA box binding protein |
| 98 | Tgfb1 | Mm01178820_m1 | Transforming growth factor, beta 1 |
| **99** | **Tgfb2** | **Mm00436955_m1** | **Transforming growth factor, beta 2** |
| 100 | Tgfbr1 | Mm00436964_m1 | Transforming growth factor, beta receptor I |
| 101 | Tgfbr2 | Mm00436977_m1 | Transforming growth factor, beta receptor II |
| **102** | **Tlr2** | **Mm00442346_m1** | **Toll-like receptor 2** |
| 103 | Tlr3 | Mm01207404_m1 | Toll-like receptor 3 |
| 104 | Tlr4 | Mm00445273_m1 | Toll-like receptor 4 |
| 105 | Tlr7 | Mm00446590_m1 | Toll-like receptor 7 |
| 106 | Tlr9 | Mm00446193_m1 | Toll-like receptor 9 |
| 107 | Tnf | Mm00443258_m1 | Tumor necrosis factor |
| 108 | Tnfrsf1a | Mm00441875_m1 | Tumor necrosis factor receptor superfamily, member 1a |
| 109 | Tnfrsf1b | Mm00441889_m1 | Tumor necrosis factor receptor superfamily, member 1b |
| 110 | *Ubc* | Mm01201237_m1 | Ubiquitin C |
| 111 | Xcl1 | Mm00434772_m1 | Chemokine (C motif) ligand 1 |
| 112 | Xcr1 | Mm00442206_s1 | Chemokine (C motif) receptor 1 |

Classical reference genes according to literature are indicated in Italics. Bold letters indicate genes with missing qPCR data*.*
