# Supplementary material for: The Challenge of Stability in High-Throughput Gene Expression Analysis: Comprehensive Selection and Evaluation of Reference Genes for BALB/c Mice Spleen Samples in the Leishmania infantum Infection Model
Source: PLoS One. 2016 Sep 26;11(9):e0163219. doi: 10.1371/journal.pone.0163219 (PMC5036817; doi:10.1371/journal.pone.0163219)
Supplement: S2 Table — (DOCX) [file pone.0163219.s002.docx]

**S2 Table. Stability values of 71 candidate reference genes for spleen samples of control BALB/c mice, ranked by geNorm, NormFinder and RefFinder.**

|  | **geNorm** | | | **NormFinder** | | **RefFinder** | |
| --- | --- | --- | --- | --- | --- | --- | --- |
| **Gen name** | **Ranking** | **Stability value (M)** | **Coefficient of variation (CV)** | **Ranking** | **Stability value** | **Ranking** | **Stability value** |
| Il10rb | 1 | 0.285 | 0.279 | 15 | 0.269 | 16 | 18.50 |
| Il18bp | 2 | 0.305 | 0.207 | 8 | 0.217 | 23 | 24.19 |
| Cxcl10 | 3 | 0.319 | 0.255 | 14 | 0.259 | 41 | 32.96 |
| Myd88 | 4 | 0.334 | 0.118 | 1 | 0.111 | 3 | 5.14 |
| *Hprt* | 5 | 0.348 | 0.225 | 7 | 0.209 | 1 | 3.51 |
| Il13ra1 | 6 | 0.364 | 0.216 | 12 | 0.243 | 27 | 26.70 |
| Il2rg | 7 | 0.379 | 0.185 | 2 | 0.175 | 8 | 11.61 |
| Il10ra | 8 | 0.389 | 0.198 | 6 | 0.207 | 4 | 5.58 |
| Itgb2 | 9 | 0.399 | 0.183 | 3 | 0.176 | 6 | 7.47 |
| Stat1 | 10 | 0.407 | 0.244 | 13 | 0.258 | 11 | 16.29 |
| Tgfbr1 | 11 | 0.417 | 0.193 | 5 | 0.206 | 15 | 17.08 |
| Il6ra | 12 | 0.425 | 0.244 | 11 | 0.238 | 12 | 16.49 |
| Stat6 | 13 | 0.432 | 0.209 | 4 | 0.194 | 7 | 9.32 |
| Ccr4 | 14 | 0.437 | 0.249 | 9 | 0.235 | 9 | 14.59 |
| Il18 | 15 | 0.443 | 0.320 | 24 | 0.296 | 19 | 20.69 |
| Tgfb1 | 16 | 0.449 | 0.260 | 10 | 0.237 | 31 | 28.43 |
| Ifngr1 | 17 | 0.455 | 0.282 | 31 | 0.330 | 56 | 45.17 |
| Tnfrsf1b | 18 | 0.461 | 0.270 | 25 | 0.301 | 24 | 24.32 |
| Il2rb | 19 | 0.466 | 0.290 | 28 | 0.317 | 49 | 40.34 |
| Stat3 | 20 | 0.471 | 0.300 | 22 | 0.291 | 40 | 31.34 |
| Stat5a | 21 | 0.477 | 0.332 | 33 | 0.343 | 46 | 37.49 |
| Cxcr2 | 22 | 0.481 | 0.311 | 34 | 0.347 | 44 | 34.44 |
| Cxcr3 | 23 | 0.486 | 0.261 | 18 | 0.271 | 17 | 19.06 |
| Il6st | 24 | 0.491 | 0.265 | 20 | 0.286 | 28 | 27.29 |
| Cxcr4 | 25 | 0.496 | 0.339 | 30 | 0.328 | 29 | 27.53 |
| Icos | 26 | 0.502 | 0.299 | 17 | 0.270 | 18 | 20.34 |
| Stat4 | 27 | 0.507 | 0.295 | 21 | 0.288 | 2 | 4.92 |
| Tlr4 | 28 | 0.511 | 0.283 | 16 | 0.270 | 14 | 17.02 |
| Ccl22 | 29 | 0.516 | 0.312 | 23 | 0.296 | 26 | 26.25 |
| Il17ra | 30 | 0.521 | 0.302 | 19 | 0.271 | 10 | 15.02 |
| Tnf | 31 | 0.526 | 0.306 | 26 | 0.310 | 20 | 20.72 |
| *Ubc* | 32 | 0.531 | 0.305 | 27 | 0.314 | 13 | 16.87 |
| Il1rn | 33 | 0.536 | 0.411 | 37 | 0.374 | 50 | 40.53 |
| Xcl1 | 34 | 0.541 | 0.330 | 32 | 0.340 | 30 | 27.69 |
| Cd86 | 35 | 0.547 | 0.403 | 36 | 0.365 | 5 | 6.64 |
| Ccr5 | 36 | 0.552 | 0.306 | 29 | 0.320 | 42 | 32.98 |
| *B2m* | 37 | 0.558 | 0.343 | 38 | 0.374 | 34 | 29.05 |
| Ccr2 | 38 | 0.564 | 0.375 | 35 | 0.360 | 33 | 28.96 |
| Il1b | 39 | 0.569 | 0.412 | 40 | 0.392 | 55 | 43.74 |
| Ccr1 | 40 | 0.575 | 0.341 | 39 | 0.391 | 21 | 22.21 |
| Cd54 | 41 | 0.580 | 0.348 | 42 | 0.405 | 62 | 56.04 |
| Stat5b | 42 | 0.586 | 0.343 | 43 | 0.408 | 54 | 42.83 |
| Ccr7 | 43 | 0.591 | 0.385 | 47 | 0.428 | 57 | 45.56 |
| Il4ra | 44 | 0.596 | 0.360 | 49 | 0.440 | 64 | 57.40 |
| Tgfbr2 | 45 | 0.602 | 0.406 | 53 | 0.465 | 61 | 49.29 |
| Ptges | 46 | 0.608 | 0.382 | 48 | 0.437 | 58 | 47.68 |
| Il12rb2 | 47 | 0.613 | 0.439 | 45 | 0.415 | 52 | 41.17 |
| Il1a | 48 | 0.619 | 0.421 | 46 | 0.421 | 25 | 25.90 |
| Gata3 | 49 | 0.625 | 0.384 | 41 | 0.404 | 59 | 47.69 |
| Il5ra | 50 | 0.631 | 0.436 | 44 | 0.412 | 51 | 40.95 |
| Il1r1 | 51 | 0.637 | 0.519 | 50 | 0.440 | 38 | 30.54 |
| Tlr9 | 52 | 0.643 | 0.482 | 51 | 0.440 | 36 | 29.35 |
| Cd80 | 53 | 0.650 | 0.460 | 52 | 0.452 | 45 | 34.99 |
| Tlr3 | 54 | 0.656 | 0.531 | 55 | 0.476 | 35 | 29.27 |
| Il18r1 | 55 | 0.662 | 0.404 | 54 | 0.469 | 60 | 48.96 |
| Tlr7 | 56 | 0.668 | 0.484 | 56 | 0.488 | 48 | 39.77 |
| *Pgk1* | 57 | 0.676 | 0.633 | 57 | 0.509 | 63 | 56.21 |
| Il27ra | 58 | 0.683 | 0.482 | 58 | 0.553 | 65 | 57.64 |
| Icam2 | 59 | 0.692 | 0.462 | 60 | 0.584 | 66 | 60.93 |
| Ccl5 | 60 | 0.700 | 0.555 | 61 | 0.591 | 70 | 66.23 |
| Il21r | 61 | 0.708 | 0.513 | 62 | 0.610 | 69 | 65.23 |
| Ifngr2 | 62 | 0.715 | 0.499 | 63 | 0.626 | 68 | 64.17 |
| Icosl | 63 | 0.723 | 0.652 | 59 | 0.563 | 37 | 30.47 |
| Itgal | 64 | 0.732 | 0.565 | 67 | 0.657 | 67 | 63.71 |
| Il2ra | 65 | 0.742 | 0.683 | 65 | 0.640 | 39 | 30.82 |
| Il12a | 66 | 0.752 | 0.771 | 64 | 0.635 | 47 | 38.41 |
| *Polr2a* | 67 | 0.761 | 0.724 | 66 | 0.640 | 53 | 41.20 |
| *Tbp* | 68 | 0.775 | 0.823 | 68 | 0.786 | 22 | 23.68 |
| Cxcl9 | 69 | 0.790 | 0.796 | 70 | 0.826 | 32 | 28.47 |
| Xcr1 | 70 | 0.803 | 0.927 | 69 | 0.824 | 43 | 34.22 |
| Tnfrsf1a | 71 | 0.821 | 0.575 | 71 | 0.926 | 71 | 71.00 |

Classical reference genes according to literature are indicated in Italics.
