# Supplementary material for: The Challenge of Stability in High-Throughput Gene Expression Analysis: Comprehensive Selection and Evaluation of Reference Genes for BALB/c Mice Spleen Samples in the Leishmania infantum Infection Model
Source: PLoS One. 2016 Sep 26;11(9):e0163219. doi: 10.1371/journal.pone.0163219 (PMC5036817; doi:10.1371/journal.pone.0163219)
Supplement: S3 Table — (DOCX) [file pone.0163219.s003.docx]

**S3 Table. Stability values of 71 candidate reference genes for spleen samples of *Leishmania*-infected BALB/c mice, ranked by geNorm, NormFinder and RefFinder.**

|  | **geNorm** | | | **NormFinder** | | **RefFinder** | |
| --- | --- | --- | --- | --- | --- | --- | --- |
| **Gen name** | **Ranking** | **Stability value (M)** | **Coefficient of variation (CV)** | **Ranking** | **Stability value** | **Ranking** | **Stability value** |
| Il6st | 1 | 0.251 | 0.196 | 5 | 0.188 | 5 | 8.97 |
| Itgb2 | 2 | 0.253 | 0.141 | 1 | 0.134 | 3 | 5.63 |
| Il10rb | 3 | 0.268 | 0.191 | 6 | 0.191 | 1 | 3.45 |
| Tgfbr1 | 4 | 0.292 | 0.190 | 8 | 0.196 | 2 | 4.49 |
| Tgfb1 | 5 | 0.298 | 0.196 | 9 | 0.221 | 9 | 12.52 |
| Stat6 | 6 | 0.313 | 0.159 | 3 | 0.165 | 4 | 7.00 |
| Il2rg | 7 | 0.326 | 0.158 | 2 | 0.159 | 7 | 9.98 |
| *Hprt* | 8 | 0.336 | 0.199 | 7 | 0.194 | 35 | 30.20 |
| Il18 | 9 | 0.346 | 0.220 | 13 | 0.228 | 6 | 9.03 |
| Il10ra | 10 | 0.359 | 0.198 | 10 | 0.224 | 12 | 15.16 |
| Il4ra | 11 | 0.371 | 0.323 | 22 | 0.326 | 44 | 34.10 |
| Ifngr1 | 12 | 0.384 | 0.335 | 27 | 0.347 | 30 | 26.41 |
| Stat5a | 13 | 0.396 | 0.341 | 29 | 0.351 | 51 | 40.02 |
| Ccl5 | 14 | 0.406 | 0.328 | 37 | 0.371 | 59 | 48.62 |
| *Pgk1* | 15 | 0.415 | 0.236 | 14 | 0.241 | 10 | 12.64 |
| Il6ra | 16 | 0.422 | 0.255 | 11 | 0.225 | 15 | 18.80 |
| Il2rb | 17 | 0.432 | 0.367 | 35 | 0.367 | 46 | 35.07 |
| Cxcr4 | 18 | 0.441 | 0.432 | 38 | 0.374 | 50 | 38.77 |
| Stat3 | 19 | 0.449 | 0.322 | 19 | 0.314 | 53 | 42.91 |
| Myd88 | 20 | 0.458 | 0.209 | 4 | 0.186 | 8 | 10.32 |
| Il13ra1 | 21 | 0.466 | 0.344 | 33 | 0.359 | 48 | 36.81 |
| Il17ra | 22 | 0.474 | 0.230 | 12 | 0.226 | 11 | 15.01 |
| Tlr4 | 23 | 0.483 | 0.281 | 15 | 0.249 | 13 | 15.31 |
| Stat4 | 24 | 0.491 | 0.265 | 17 | 0.304 | 17 | 20.32 |
| Ccr2 | 25 | 0.5 | 0.320 | 18 | 0.304 | 14 | 15.43 |
| Gata3 | 26 | 0.507 | 0.344 | 16 | 0.298 | 20 | 21.99 |
| Cd86 | 27 | 0.517 | 0.325 | 20 | 0.318 | 26 | 25.35 |
| Cxcr3 | 28 | 0.525 | 0.330 | 26 | 0.340 | 31 | 26.56 |
| Il1r1 | 29 | 0.532 | 0.367 | 31 | 0.356 | 21 | 22.71 |
| Cd80 | 30 | 0.539 | 0.362 | 25 | 0.337 | 43 | 32.60 |
| Ccr5 | 31 | 0.545 | 0.351 | 23 | 0.329 | 18 | 20.71 |
| *Ubc* | 32 | 0.551 | 0.355 | 28 | 0.350 | 28 | 25.71 |
| Icos | 33 | 0.556 | 0.336 | 21 | 0.326 | 16 | 18.91 |
| Il12rb2 | 34 | 0.562 | 0.370 | 30 | 0.352 | 38 | 31.20 |
| Ccl22 | 35 | 0.568 | 0.417 | 34 | 0.367 | 40 | 31.65 |
| Il5ra | 36 | 0.573 | 0.358 | 36 | 0.370 | 25 | 24.93 |
| Tnf | 37 | 0.578 | 0.355 | 32 | 0.359 | 22 | 23.21 |
| Il18bp | 38 | 0.583 | 0.336 | 24 | 0.332 | 24 | 23.52 |
| *B2m* | 39 | 0.588 | 0.346 | 45 | 0.421 | 19 | 21.06 |
| Ccr1 | 40 | 0.593 | 0.408 | 42 | 0.413 | 37 | 31.05 |
| *Polr2a* | 41 | 0.598 | 0.414 | 43 | 0.418 | 36 | 30.45 |
| Tlr9 | 42 | 0.604 | 0.429 | 47 | 0.429 | 29 | 25.93 |
| Il1b | 43 | 0.609 | 0.434 | 41 | 0.402 | 32 | 27.67 |
| Il18r1 | 44 | 0.615 | 0.343 | 39 | 0.388 | 55 | 46.71 |
| Cxcr2 | 45 | 0.621 | 0.350 | 40 | 0.394 | 58 | 48.46 |
| Tnfrsf1b | 46 | 0.626 | 0.371 | 44 | 0.420 | 54 | 45.12 |
| Ccr7 | 47 | 0.631 | 0.457 | 48 | 0.447 | 52 | 42.69 |
| Icosl | 48 | 0.636 | 0.487 | 50 | 0.471 | 34 | 29.07 |
| Ccr4 | 49 | 0.641 | 0.418 | 46 | 0.425 | 57 | 47.98 |
| Il1a | 50 | 0.647 | 0.501 | 49 | 0.460 | 42 | 32.24 |
| Cd54 | 51 | 0.655 | 0.436 | 51 | 0.487 | 62 | 52.76 |
| Il12a | 52 | 0.663 | 0.600 | 56 | 0.553 | 39 | 31.63 |
| Tlr3 | 53 | 0.671 | 0.494 | 52 | 0.519 | 27 | 25.64 |
| Tgfbr2 | 54 | 0.679 | 0.468 | 55 | 0.534 | 63 | 54.77 |
| Il27ra | 55 | 0.687 | 0.528 | 57 | 0.554 | 65 | 59.98 |
| Xcl1 | 56 | 0.696 | 0.534 | 54 | 0.534 | 49 | 38.52 |
| Stat1 | 57 | 0.704 | 0.544 | 53 | 0.530 | 60 | 51.79 |
| Stat5b | 58 | 0.712 | 0.478 | 58 | 0.570 | 66 | 62.47 |
| Tlr7 | 59 | 0.721 | 0.505 | 60 | 0.584 | 33 | 28.55 |
| Il1rn | 60 | 0.73 | 0.558 | 59 | 0.571 | 56 | 47.79 |
| Itgal | 61 | 0.74 | 0.518 | 61 | 0.648 | 69 | 65.49 |
| Ptges | 62 | 0.75 | 0.502 | 62 | 0.652 | 67 | 63.96 |
| Ifngr2 | 63 | 0.76 | 0.557 | 65 | 0.682 | 64 | 59.68 |
| Tnfrsf1a | 64 | 0.77 | 0.541 | 64 | 0.681 | 68 | 65.21 |
| Il2ra | 65 | 0.779 | 0.669 | 63 | 0.663 | 45 | 34.58 |
| *Tbp* | 66 | 0.79 | 0.658 | 66 | 0.708 | 23 | 23.42 |
| Il21r | 67 | 0.801 | 0.650 | 68 | 0.759 | 70 | 68.74 |
| Icam2 | 68 | 0.813 | 0.595 | 69 | 0.793 | 71 | 69.25 |
| Cxcl10 | 69 | 0.825 | 1.044 | 67 | 0.738 | 61 | 52.29 |
| Xcr1 | 70 | 0.838 | 0.797 | 70 | 0.824 | 41 | 31.85 |
| Cxcl9 | 71 | 0.86 | 0.980 | 71 | 1.045 | 47 | 36.58 |

Classical reference genes according to literature are indicated in Italics.
