# Supplementary material for: The Challenge of Stability in High-Throughput Gene Expression Analysis: Comprehensive Selection and Evaluation of Reference Genes for BALB/c Mice Spleen Samples in the Leishmania infantum Infection Model
Source: PLoS One. 2016 Sep 26;11(9):e0163219. doi: 10.1371/journal.pone.0163219 (PMC5036817; doi:10.1371/journal.pone.0163219)
Supplement: S4 Table — (DOCX) [file pone.0163219.s004.docx]

**S4 Table. Stability values of 71 candidate reference genes for spleen samples of control and *Leishmania*-infected BALB/c mice,** **ranked by geNorm, NormFinder and RefFinder.**

|  | **geNorm** | | | **NormFinder** | | **RefFinder** | |
| --- | --- | --- | --- | --- | --- | --- | --- |
| **Gen name** | **Ranking** | **Stability value (M)** | **Coefficient of variation (CV)** | **Ranking** | **Stability value** | **Ranking** | **Stability value** |
| Itgb2 | 1 | 0.293 | 0.172 | 6 | 0.092 | 1 | 2.43 |
| Stat6 | 2 | 0.307 | 0.199 | 9 | 0.111 | 2 | 4.25 |
| Il6st | 3 | 0.321 | 0.264 | 31 | 0.160 | 19 | 19.83 |
| Il2rg | 4 | 0.351 | 0.171 | 1 | 0.060 | 3 | 6.03 |
| Il10ra | 5 | 0.370 | 0.201 | 8 | 0.102 | 4 | 7.54 |
| Tgfbr1 | 6 | 0.389 | 0.230 | 35 | 0.174 | 9 | 12.16 |
| *Hprt* | 7 | 0.400 | 0.230 | 23 | 0.137 | 6 | 9.90 |
| Tgfb1 | 8 | 0.409 | 0.279 | 45 | 0.193 | 18 | 19.01 |
| Il10rb | 9 | 0.416 | 0.255 | 22 | 0.129 | 8 | 12.12 |
| Il6ra | 10 | 0.426 | 0.247 | 2 | 0.073 | 16 | 16.79 |
| Myd88 | 11 | 0.433 | 0.197 | 14 | 0.120 | 5 | 7.68 |
| Il18 | 12 | 0.440 | 0.273 | 4 | 0.082 | 7 | 10.49 |
| Stat3 | 13 | 0.452 | 0.311 | 12 | 0.115 | 36 | 30.86 |
| Ifngr1 | 14 | 0.463 | 0.323 | 40 | 0.190 | 43 | 34.09 |
| Stat5a | 15 | 0.471 | 0.333 | 11 | 0.111 | 34 | 30.55 |
| Il13ra1 | 16 | 0.479 | 0.301 | 51 | 0.207 | 37 | 31.28 |
| Tnfrsf1b | 17 | 0.487 | 0.323 | 32 | 0.164 | 35 | 30.78 |
| Il2rb | 18 | 0.493 | 0.334 | 16 | 0.123 | 32 | 29.84 |
| Il4ra | 19 | 0.499 | 0.345 | 24 | 0.137 | 57 | 47.00 |
| Cxcr2 | 20 | 0.504 | 0.339 | 37 | 0.180 | 51 | 39.15 |
| Cxcr4 | 21 | 0.509 | 0.394 | 47 | 0.199 | 28 | 28.05 |
| Il17ra | 22 | 0.517 | 0.279 | 19 | 0.128 | 11 | 13.09 |
| Tlr4 | 23 | 0.525 | 0.279 | 3 | 0.076 | 12 | 14.19 |
| Stat4 | 24 | 0.531 | 0.277 | 5 | 0.085 | 10 | 12.75 |
| Icos | 25 | 0.541 | 0.323 | 21 | 0.128 | 15 | 16.79 |
| Ccr2 | 26 | 0.550 | 0.345 | 7 | 0.095 | 14 | 16.69 |
| Cd86 | 27 | 0.558 | 0.368 | 18 | 0.127 | 13 | 16.66 |
| Cxcr3 | 28 | 0.564 | 0.336 | 42 | 0.193 | 20 | 22.09 |
| Ccr4 | 29 | 0.570 | 0.340 | 38 | 0.183 | 53 | 41.46 |
| *Ubc* | 30 | 0.576 | 0.333 | 15 | 0.121 | 17 | 18.37 |
| Ccl22 | 31 | 0.581 | 0.368 | 34 | 0.173 | 29 | 28.21 |
| Gata3 | 32 | 0.586 | 0.366 | 17 | 0.124 | 46 | 34.54 |
| Il18bp | 33 | 0.592 | 0.369 | 58 | 0.244 | 30 | 29.14 |
| Tnf | 34 | 0.599 | 0.361 | 43 | 0.193 | 23 | 25.13 |
| Il1b | 35 | 0.606 | 0.424 | 27 | 0.152 | 42 | 32.22 |
| Ccr1 | 36 | 0.612 | 0.383 | 28 | 0.152 | 22 | 24.33 |
| Cd80 | 37 | 0.619 | 0.410 | 10 | 0.111 | 41 | 32.13 |
| Il1r1 | 38 | 0.624 | 0.507 | 55 | 0.229 | 25 | 25.72 |
| Ccr5 | 39 | 0.630 | 0.413 | 67 | 0.280 | 38 | 31.48 |
| Il12rb2 | 40 | 0.636 | 0.423 | 56 | 0.233 | 50 | 38.44 |
| *Pgk1* | 41 | 0.641 | 0.520 | 26 | 0.145 | 54 | 43.64 |
| *B2m* | 42 | 0.646 | 0.388 | 57 | 0.233 | 26 | 26.49 |
| Il1a | 43 | 0.651 | 0.458 | 20 | 0.128 | 31 | 29.28 |
| Tlr9 | 44 | 0.657 | 0.462 | 50 | 0.204 | 24 | 25.63 |
| Il5ra | 45 | 0.662 | 0.469 | 64 | 0.265 | 52 | 39.95 |
| Il18r1 | 46 | 0.667 | 0.372 | 13 | 0.120 | 55 | 44.26 |
| Cd54 | 47 | 0.673 | 0.390 | 29 | 0.159 | 62 | 54.38 |
| Ccr7 | 48 | 0.678 | 0.446 | 59 | 0.248 | 58 | 49.94 |
| Ccl5 | 49 | 0.685 | 0.479 | 30 | 0.159 | 65 | 59.16 |
| Tgfbr2 | 50 | 0.691 | 0.439 | 49 | 0.200 | 61 | 52.89 |
| Stat5b | 51 | 0.696 | 0.411 | 46 | 0.197 | 63 | 55.64 |
| Icosl | 52 | 0.703 | 0.585 | 33 | 0.167 | 33 | 30.27 |
| Xcl1 | 53 | 0.709 | 0.542 | 62 | 0.256 | 48 | 34.99 |
| Il1rn | 54 | 0.715 | 0.516 | 36 | 0.176 | 56 | 44.41 |
| *Polr2a* | 55 | 0.722 | 0.604 | 25 | 0.141 | 49 | 38.35 |
| Il27ra | 56 | 0.729 | 0.507 | 52 | 0.208 | 64 | 58.09 |
| Ptges | 57 | 0.736 | 0.440 | 53 | 0.210 | 66 | 60.22 |
| Tlr3 | 58 | 0.743 | 0.558 | 68 | 0.283 | 40 | 31.97 |
| Stat1 | 59 | 0.751 | 0.656 | 71 | 0.372 | 59 | 51.17 |
| Tlr7 | 60 | 0.758 | 0.535 | 61 | 0.252 | 45 | 34.18 |
| Il12a | 61 | 0.765 | 0.677 | 44 | 0.193 | 44 | 34.16 |
| Cxcl10 | 62 | 0.773 | 1.041 | 66 | 0.278 | 60 | 52.14 |
| Il2ra | 63 | 0.782 | 0.672 | 48 | 0.200 | 27 | 27.22 |
| Itgal | 64 | 0.791 | 0.540 | 39 | 0.189 | 68 | 64.74 |
| Ifngr2 | 65 | 0.799 | 0.542 | 63 | 0.258 | 67 | 64.21 |
| Il21r | 66 | 0.809 | 0.579 | 60 | 0.249 | 70 | 67.74 |
| Icam2 | 67 | 0.818 | 0.536 | 69 | 0.283 | 69 | 66.74 |
| *Tbp* | 68 | 0.829 | 0.741 | 41 | 0.191 | 21 | 23.68 |
| Tnfrsf1a | 69 | 0.841 | 0.552 | 54 | 0.213 | 71 | 69.49 |
| Xcr1 | 70 | 0.855 | 0.857 | 65 | 0.273 | 39 | 31.85 |
| Cxcl9 | 71 | 0.874 | 1.108 | 70 | 0.365 | 47 | 34.59 |

Classical reference genes according to literature are indicated in Italics.
